# Effects of Instrumental, Manipulative and Soft Tissue Approaches for the Suboccipital Region in Subjects with Chronic Mechanical Neck Pain. A Randomized Controlled Trial

**DOI:** 10.3390/ijerph18168636

**Published:** 2021-08-16

**Authors:** Juan José Arjona Retamal, Alejandro Fernández Seijo, José David Torres Cintas, Ana I. de-la-Llave-Rincón, Andrea Caballero Bragado

**Affiliations:** 1 Faculty of Physiotherapy, King Juan Carlos University, 28922 Madrid, Spain; alex_el_leones@hotmail.com (A.F.S.); davidtorrescintas@gmail.com (J.D.T.C.); 2 Department of Physical Therapy, Occupational Therapy, Rehabilitation and Physical Medicine, Universidad Rey Juan Carlos, 28922 Madrid, Spain; anaisabel.delallave@urjc.es; 3 Faculty of Psychology, Autonomous University of Madrid, 28049 Madrid, Spain; andrea_cocker@hotmail.com

**Keywords:** cervical pain, suboccipital muscles, trigger points, INYBI, cervical manipulation

## Abstract

The INYBI is an instrument used to release the suboccipital myofascial area. There is scarce evidence of its efficacy. A randomized controlled, double-blinded, longitudinal and prospective trial was performed. Ninety-six subjects (aged 29.47 ± 5.16 years) (70 women) with chronic neck pain were randomly assigned to the manual suboccipital inhibition technique (MSIT), instrumental suboccipital inhibition (INYBI) or the INYBI plus upper cervical manipulation technique (INYBI + UCMT) groups and received two sessions with a week interval between them. The Neck Disability Index was used before the first intervention and two weeks after the second intervention. Pre- and post-measurements were taken on both intervention days for pressure pain threshold of the upper trapezius and suboccipital muscles, self-perceived pain and cervical range of motion. In spite of a significant general improvement in time that was found for the three groups for all of the outcome measurements (*p* < 0.05 in all cases), no between-groups differences were found (*p* > 0.05 in all cases), with the exception of self-perceived pain for left rotation (*p* = 0.024), with the MSIT group showing the lower improvement. However, the higher degree of within-group improvements was found for the INYBI + UCMT group. It was concluded that the myofascial release therapy in the suboccipital area is effective in patients with chronic neck pain, either through a manual application or by means of the INYBI tool. Moreover, the addition of craniocervical manipulation achieved the higher within-group improvements, but with no statistical significance.

## 1. Introduction

Cervicalgia is a health and social issue [1]. It limits the autonomy and quality of life of those who suffer it [1], being the world’s fourth cause of disability, with an annual prevalence rate ranging between 30 and 50% in the global population [2]. Its economic consequences include health care costs, a decrease in labor productivity, work absenteeism and compensation [3]. It tends to become a chronic problem, since close to 50% of people will continue to have pain after a cervicalgia episode [1,2].

Patients with chronic neck pain (CNP) exhibit diminished neck range of motion [4] and hyperalgesia, with higher mechanical sensitivity, in several tissues such as nerve trunks [5] and neck muscles [4,6]. These facts have been explained by peripheral sensitization [7]. The persistence of the nociceptive afferences due to peripheral facilitation might induce central sensitization [6,8]. Due to postural [9,10], mechanical [11] and neurological [12] factors, the suboccipital region has been frequently targeted when treating patients with craniocervical pain disorders [13,14,15].

Thus, in order to manage spinal pain, myofascial release therapy (MRT) and spinal manipulation (SM) are frequently used. Regarding MRT, it implies the application of a low load, long duration force which has classically attempted to achieve its goal by stretching the connective tissues with the aim of improving their properties and restoring their optimal length, and thereby diminishing pain and increasing range of motion [16,17]. Since the application of MRT can be tiring or even painful for the therapist, several instruments have been used to facilitate it. With respect to the upper neck area, this was the case for tools such as a triangle-shaped pillow [18], INYBI (Eskua Health Technologies S.L., Donostia, Spain) [19] and inflatable and massage balls [20]. Specifically, the INYBI is an instrument which has been recently developed to self-release the suboccipital muscles. It has three different heads which differ in the hardness. It can be used in three different positions, to adapt to the patient’s neck curvature. It also has a vibration option with different frequencies. The INYBI has previously shown to be almost as effective as manual MRT in CNP patients in the short term [19]. However, to date, no research has been performed to assess its medium-term effects in CNP.

On the other hand, spinal manipulation is also a commonly used intervention for pain disorders. Several mechanisms are thought to be implied in the way that spinal manipulation works, and some of them could be done extensively with MRT [21]. It uses a mechanical stimulus in order to trigger several neurophysiological responses, which may include hypoalgesia [22], sympathetic responses, neuromuscular adaptations [23,24,25], kinesthetic sensibility improvements [26] and changes in biomarkers levels [27,28].

In general terms, physiotherapy treatments, and especially manual therapy interventions, are not usually composed by the application of isolated techniques, but by a sum of them. Some research has shown that the addition of techniques is more effective than any of them on their own in craniocervical disorders [14,15,29]. Despite that self-MRT can be performed by patients at home, it could also be used at physiotherapy and rehabilitation centers [19], for instance as a preparatory intervention combined with other kinds of therapy, such as manipulative therapy. However, to the authors’ knowledge, there is no research about the benefits of the combination of INYBI self-MRT and upper cervical manipulation in CNP patients. Therefore, the aim of this study is to compare the effectiveness between the manual suboccipital inhibition technique (MSIT), the self-MRT carried out with the INYBI instrument and the combination of INYBI plus the upper cervical manipulation technique (UCMT) in patients with CNP. It was hypothesized that the combined treatment would obtain the better results.

## 2. Material and Methods

### 2.1. Design

A randomized, double-blinded, controlled trial (ClinicalTrials.gov; registration number NCT04777890) was performed. The study received the approval by the Research Ethics Committee of the Universidad Rey Juan Carlos (Madrid, Spain) (0312201815318) and was conducted following the ethical guidelines of the Declaration of Helsinki.

### 2.2. Participants

**Figure 1:**
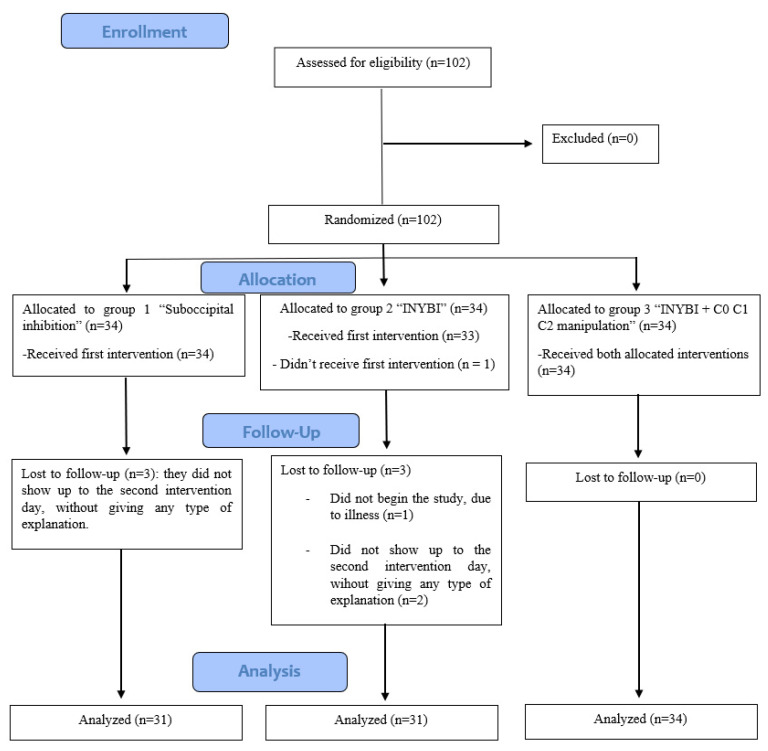
Flowchart of study participants according to CONSORT.

The recruitment took place in a physiotherapy and osteopathy private center in Madrid (Spain). To be included, the patients between 18 and 40 years old had to suffer from mechanical CNP with an evolution of at least 3 months, showing pain increased with maintained postures, with movement and during spinal muscles palpation and that can curse with trapezius pain. Pain should be located between the occipital bone, including the cervicothoracic junction to the fourth dorsal vertebra. Subjects were excluded if they could not read and/or fill in the informed consent; presented psychological pathologies, such as depression or anxiety; had received a manual treatment two months before the beginning of the clinical trial; were in treatment with any type of analgesic, anti-inflammatory or neuromodulator medication or had used it in the previous 72 h; felt fear to vertebral manipulation in the upper cervical area; or presented contraindications to manual therapy, such as: positive Klein Test and other tests of the vertebral artery’s integrity, positive cervical instability test and positive Spurling Test; osteitis; cervical rheumatic diseases; congenital anomalies such as Arnold Chiari or Klippel-Feil; hemorrhages; recent cranial bone fracture at its base; tumors; previous cranial bone or cervical surgery; platybasia; upper cervical osteoarthritis; pathologies that weaken in a significant way the bone system, such as severe osteoporosis, bone metastases; instability signs in the upper cervical region; cervicobrachial neuralgia; diabetes mellitus or any arm/shoulder joint pathologies. A total of 102 participants were randomized because all of them agreed to participate in the study, but one finally decided not to do so and five just completed the first phase and did not receive the second intervention [30,31] (Figure 1).

### 2.3. Protocol

The recruiter collected the participants’ informed consent and demographic data (age, sex), besides relevant clinical information related to the study criteria. A different examiner carried out the pre-intervention assessment. After it, the first intervention was applied by another researcher, and followed by the first post-treatment evaluation, which was undertaken immediately after the intervention (and carried out by the same pre-intervention examiner). One week later, a second session was performed, including the same procedure related to pre- and post-interventions assessment. With respect to the Neck Disability Index (NDI), it was only filled in before the first intervention (in the clinic) and 15 days after the second intervention (the recruiter sent it to the patient via e-mail). The instructions were included in the questionnaire. 

### 2.4. Outcome Measures

#### 2.4.1. Cervical Range of Motion (ROM)

The measurement of the cervical ROM was carried out with the goniometer CROM^®^ (Performance Attainment Associates, St Paul, MN, USA). It is constituted by a floating compass which is attached to the head with Velcro straps. This tool has shown an intratester reliability of 0.87–0.96, and a standard error of measurement of 2.3°–4.1° [32]. During the measurement, the patient was seated upright. The neck ROM was measured in every plane. 

#### 2.4.2. Self-Perceived Neck Pain

Neck pain was evaluated with a visual analog scale (VAS). It consists of a horizontal 10 cm line where the participant has to mark the intensity of the pain he/she perceives during the movements of the neck in every plane [33]. A mark of 0 means no pain at all, while 10 means the worst possible pain. The mark was scored to the nearest millimeter [34]. This scale has shown to be effective, sensitive and appropriate in order to measure acute and chronic pain intensity [35,36] with an excellent test-retest reliability (ICC 0.92) [37].

#### 2.4.3. Pressure Pain Thresholds

To assess the sensitization of the suboccipital (SM) and upper trapezius (UT) muscles, their pressure pain thresholds (PPT), which is the minimum amount of pressure needed to evoke discomfort or pain [38], were measured. The location of the tense bands in these muscles has been previously described [39]. A model FPX 25 digital algometer (Wagner Instruments, Greenwich, CT) was used. It has a 1 cm^2^ contact area, which was applied perpendicularly with an increasing pressure of 1 kg/cm^2^ per second approximately. With the patient seated upright, each PPT was measured 3 times, taking into account the arithmetic mean of the three of them. All the participants were instructed in the same way to warn when the sensation of pressure became uncomfortable or painful.

#### 2.4.4. NDI

The NDI is the most used cervical pain disability scale to assess the functional level of the patients [40]. It is made up of 10 items, each scoring between 0 and 5 points, 50 being the maximum score and 0 the minimum score [14,15]. The bigger the score, the higher the disability level. 

### 2.5. Interventions

In order to distribute the sample in three intervention groups (one control group and two experimental groups), Microsoft Excel (Microsoft, Redmond, WA, USA) was used as the randomization method. The concealment was guaranteed by the participation of an independent collaborator, who guarded the randomization sequence. To implement random allocation, sequentially numbered opaque sealed envelopes were used. Those researchers involved in recruitment were unaware of the number sequence and the assignment to the intervention groups. As well, the interventions were blinded for the patients, recruiter and examiner, since patients were randomly allocated into the groups, and none of them knew the characteristics of the interventions and which treatment they were going to receive.

**Figure 2:**
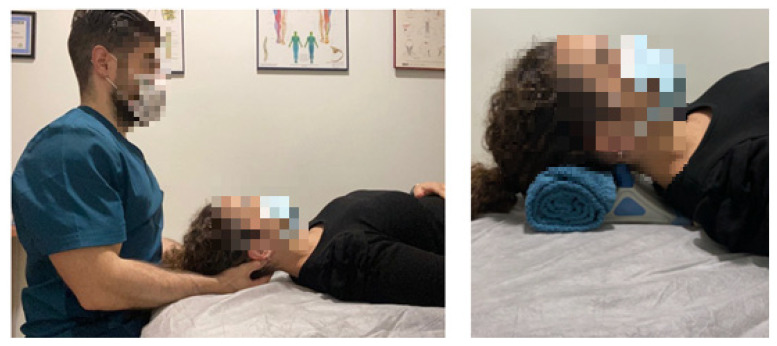
Manual suboccipital inhibition technique (**left picture**) and application of the INYBI (**right picture**).

In the three intervention groups, the participant lied in a decubitus supine position, and all the interventions were carried out by the same therapist. Participants in the control group were treated with the manual SIT (MSIT group) during a 10 min period. This procedure was undertaken according to the literature [41,42,43]. The therapist was seated at the head of the table and placed his middle and ring fingers between the occipital condyles and the spinal process of the second cervical vertebra. Then, he performed a constant pressure while keeping his metacarpophalangeal joints in 90° flexion, taking care of not being painful. A cranial mild traction was exerted also in order to increase the stretching (Figure 2, left picture). We used the MSIT as the intervention used in the control group due to the large amount of literature that supports its effectiveness. 

In the INYBI group, the therapist placed the INYBI at the suboccipital area, specifically placing the fingers of the instrument at the posterior arch of the atlas [19]. All patients received the 10 min treatment with the INYBI’s hardest head with a 50 HZ vibration frequency (Figure 2, right picture). 

**Figure 3:**
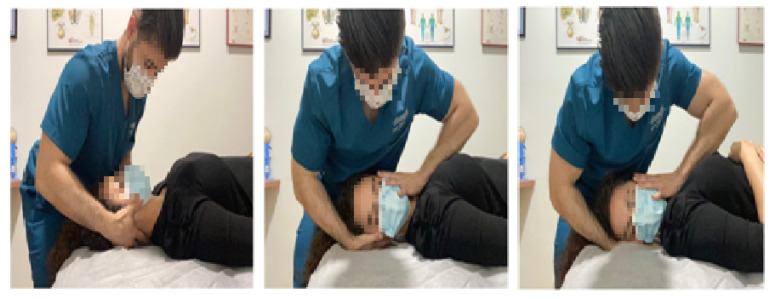
Upper cervical manipulation technique.

In the combined (INYBI + UCMT) group, the INYBI was used during a 10 min period, after which the therapist carried out the UCMT. This bilateral manipulation is a high velocity and low amplitude technique performed by cervical rotation around an imaginary vertical axis passing through the odontoid process of the axis. A mild contralateral upper cervical side-bending was added with cephalic traction and no flexion not extension parameters. The manipulative impulse was applied in rotation and attempted to increase the upper cervical joints mobility [12,44] (Figure 3).

### 2.6. Statistical Analysis

The sample size was determined with the software Granmo online v7.12 (IMIM-Hospital del Mar, Barcelona, Spain) for the variable PPT. Taking an alpha risk of 0.05 and a beta risk of 0/2 in a bilateral contrast, 33 subjects were needed in each group in order to notice a minimum difference of 0/35 points between two groups, assuming that there are 3 groups and a standard deviation of 0/4 points. A follow-up loss rate of 15% was estimated.

The statistical analysis was carried out with the software SPSS v22 (IBM, Armonk, NY, USA). The Shapiro Wilk test was made to make sure the sample distributed normally. One-way ANOVA was made to make sure the groups distributed homogeneously when they met normality criteria; if not, the Kruskal Wallis test was carried out. For qualitative variables, the Chi-Square test was used. In order to quantify the difference interval between groups, the least squares estimation was carried out. Moreover, a repeated measured ANOVA with Bonferroni correction was carried out to quantify in the three groups the pre-intervention and immediate post-intervention of the first and second sessions, respectively, as well as a pairwise comparison according to group and time. Global clinical effects for the repeated measure analysis were estimated with the Eta squared value (η^2^), and the *p* value was of <0.05. 

## 3. Results

One hundred and two subjects accepted to participate voluntarily in the study, six of which did not complete their participation. Thus, the final sample was composed of 96 subjects: 70 women, 26 men, aged 29.47 ± 5.16 years old. Figure 1 shows the flow diagram of the sample. 

The baseline characteristics of the participants in each group are presented in Table 1. No differences were found between groups at baseline (*p* > 0.05 in all cases) but for self-perceived pain in flexion, with the worst values found in the INYBI + UCMT group (*p* = 0.017).

### Outcome Measures

The three groups showed significant improvements for the time factor for all of the outcome measures (*p* < 0.001 except for flexion and extension ROM, which was *p* < 0.05). No between-groups differences were found in any variable but for the VAS during left rotation, with the smaller improvement being obtained for the MSIT group. A large clinical effect (η^2^ > 0.14) was achieved for NDI, suboccipital algometry and self-perceived pain, while a medium clinical effect (η^2^ > 0.06) was found for trapezius algometry and ROM in rotation and side bending. A small clinical effect (η^2^ > 0.01) was obtained for the flexo-extension ROM. In the within-group analysis, even though the only statistically significant difference was found for the self-perceived pain in the left rotation, the combination of INYBI + UCMT obtained the best improvements in most of the variables measured, followed by the INYBI group (Table 2, Table 3 and Table 4).

## 4. Discussion

The aim of this study was to assess the effect of instrumental MRT for the suboccipital muscles, both in an isolated application and together with an UCMT, in subjects suffering from CNP. According to our results, the three studied interventions are able to achieve big improvements for ROM, PPT, self-perceived pain and disability. In spite of the fact that no between-groups differences were found, the best outcomes were obtained for the combination of INYBI and UCMT.

Our research is the first to study the mid-term effect of INYBI in CNP, and the first to analyze its effects when combined with another intervention. MRT implies the application of a low load, long duration force which has classically attempted to achieve its goal by stretching the connective tissues with the aim of improving their properties and restoring their optimal length, and thereby diminishing pain and increasing range of motion [15,16]. Since self-MRT can be applied both for the patients at home on their own, and at the physiotherapy centers as a preparatory intervention previous to the application of other techniques, we consider that these results are very relevant in order to manage CNP patients in several settings. However, the absence of a placebo group is a limitation of the study. Further, the administered treatments can be seen as scarce, because the usual treatments in manual therapy and rehabilitation centers use to be composed by a combination of a higher number of techniques. As well, the absence of a long-term follow-up is another limitation.

Our results support the general assumption that MRT, which used to be applied manually, tends to improve ROM and pain thresholds [17]. In the specific area of the suboccipital region, previous studies found that SMIT improved quality of life [14], posture and mechanosensitivity [45], with different results about the effect on self-perceived pain [13]. With respect to this latest issue related to VAS, it must be clarified that there are two major differences between that study and ours: in the study from Antolinos et al. [13], the manual SIT was performed only in subjects with whiplash, who also showed a much higher level of VAS at baseline than ours. Further, our results show that for several measurements of VAS, the obtained improvements are higher than the 15 mm which is considered a clinically important difference [46]. For instance, in the case of the SMIT group, that difference was achieved for VAS in extension and left side-bending. In the case of the PPT, the clinically meaningful difference is 20% [47], which the SMIT group showed for the suboccipital muscles, but not for the upper trapeziuses.

Regarding to instrumental MRT, our study also confirms that it is useful in order to improve mobility and muscle status [48]. We obtained clinically relevant results with INYBI for VAS (extension, right and left side-bending) and PPT (right and left suboccipital muscles and left upper trapezius). However, it is hard to find comparable studies to ours in the neck area. In a crossover experimental research, 30 patients with neck pain used an inflatable rubber ball and a hard massage ball to self-release the suboccipital area [20]. It was found that the inflatable ball achieved better results compatible with reduced muscle tension and discomfort. Lee et al. [49] also achieved improvements in PPT and VAS with self-MRT, but they applied it not only to the neck area, but also in other parts of the body. Further, the sample in these studies were constituted by older subjects. In young participants such as ours, SMIT and self-MRT in the suboccipital region were compared in order to analyze the effect in short hamstrings [18]. Unlike our study, they found better results for the manual application.

In relation to the previous research about INYBI [19], it is interesting to note that the sample was quite similar to ours, with the exception of age, PPTs and rotation ROM, which were a little higher in that study at baseline. Moreover, it must be remembered that they only applied one session and their follow-up was limited to 30 min. Although they found no differences between instrumental and manual SIT either, manual SIT obtained slightly better results for cervical ROM than INYBI. However, we did not find such a difference. As well, such as we have done, Perez-Martinez et al. [19] obtained similar improvements for instrumental and manual SIT in relation to sensitivity and self-perceived pain. They also found better results for INYBI with respect to vertical mouth opening that we have not evaluated.

With respect to the combination of techniques, our study abounds in indicating that the sum of techniques produces an increase in the benefits of physical therapies. Even though the INYBI + UCMT group just obtained a statistically significant effect for self-perceived pain for left rotation, it obtained the best results in our study, with clinically meaningful differences for VAS (in flexion, extension and right and left side-bending), PPT (in right and left suboccipital muscles and left upper trapezius) and ROM (left and right rotation), with an increase higher than 5 degrees [32]. To our knowledge, no studies have been found adding instrumental MRT to spinal manipulation. However, several studies have tried to clarify the benefits of the sum of techniques in CNP. This way, recent studies have evaluated the effect of adding UCMT to neck exercise protocols, confirming that the addition of craniocervical manipulations generated a higher improvement compared to isolated neck exercise protocols in ROM, VAS, NDI and PPT [15,30].

Thus, our study increases the available information supporting the usefulness of targeting the craniocervical region by means of MRT in subjects suffering CNP. This way, our study highlights that it can also be done with the help of an instrumental tool such as INYBI, with the same success than the manual intervention by a professional therapist. This fact opens the possibility to use the INYBI by the patient himself at home. Further, our study shows that neck manipulation can be added to INYBI to achieve even slightly better results. More investigation is needed to know if these strategies are still effective in the long term.

Before concluding, we find it important to talk about how the Coronavirus pandemic influenced the carrying out of the study. We had to carry out some security and hygienic measures, such as giving the patients disinfectant gel for their hands, measuring their body temperature and, in the case of the researchers, wearing bodysuits, surgical gloves, masks and face shields in order to carry out the assessments and interventions. We also had to clean and disinfect every material after use and cite one patient per hour, so that he/she did not meet any other patient. Moreover, most of the losses were due to pandemic conditions, such as fear of getting sick or virus infection. 

This was not the only limitation of the study; it lacks a long-term measurement of the effects of the interventions, and the therapist carrying out the treatments was not blinded. Nevertheless, one of the strengths of this study is that it is a randomized, double-blinded trial with the collaboration of three trained therapists. 

## 5. Conclusions

MSIT, INYBI and INYBI + UCMT are equally effective in order to improve ROM, PPT, VAS and disability in patients aged between 18 and 40 years old with chronic mechanic cervical pain. The combination of INYBI and UCMT achieves the higher degree of significant improvements, followed by the INYBI group. Further, it seems necessary to investigate more about the possible applications of the INYBI. 

## Figures and Tables

**Table 1:** Baseline Characteristics of the Participants.

Characteristics	Group 1 (Suboccipital Technique) (*n* = 31)		Group 2 (INYBI) (*n* = 31)		Group 3 (INYBI + Upper Cervical Manipulation) (*n* = 34)		
	Mean	SD (95% CI)	Mean	SD (95% CI)	Mean	SD (95% CI)	*p*
Age (years)	28.71	5.53 (26.68–30.74)	29.33	4.80 (27.54–31.12)	30.29	5.14 (28.50–32.09)	0.462 *^b^*
Sex							
Masculine (n, %)	9	9.37%	8	8.33%	9	9.37%	0.823 *^b^*
Feminine (n, %)	22	22.92%	23	23.96%	25	26.04%	
Bruxism							
Yes (n, %)	17	17.7%	14	14.6%	18	18.8%	0.720 *^a^*
No (n, %)	14	14.6%	17	17.7%	16	16.7%	
Headaches							
Yes (n, %)	28	29.2%	25	26%	23	24%	0.077 *^a^*
No (n, %)	3	3.1%	6	6.3%	11	11.5%	
Car accident							
Yes (n, %)	12	12.5%	13	13.5%	13	13.5%	0.948 *^a^*
No (n, %)	19	19.8%	18	18.8%	21	21.9%	

SD, standard deviation; CI, confidence Interval. *p ^a^*: *p* value based on the results of the Chi-squared test. *p ^b^*: *p* value based on the homogeneity test when normality criteria are met (one-way ANOVA).

**Table 2:** PRE and POST Cervical Mobility Measurements (CROM).

Variable	Measurement Time	Group 1 (Suboccipital Technique) (*n* = 31)		Group 2 (INYBI) (*n* = 31)		Group 3 (INYBI + Upper Cervical Manipulation) (*n* = 34)		*p* Value (Between-Groups Comparison)
		Mean	SD (95% CI)	Mean	SD (95% CI)	Mean	SD (95% CI)	
Flexion	PRE	59.03	11.98 (54.64–63.43)	59.90	17.03 (53.54–66.26)	55.73	11.68 (51.66–59.81)	0.452
	POS 1	61.55	10.60 (57.61–65.46)	60.90	12.43 (56.26–65.54)	57.85	11.19 (53.95–61.76)	
	POS 2	61.03	11.48 (56.82–65.24)	60.77	13.81 (55.61–65.92)	59.06	11.79 (54.95–63.17)	
	POS 3	60.71	11.28 (56.57–64.85)	60.63	14.94 (55.05–66.21)	60.12 *	11.80 (56–64.24)	
Extension	PRE	65	8.71 (61.80–68.19)	61.57	14.93 (55.99–67.14)	62.76	12.30 (58.47–67.06)	0.938
	POS 1	66.87	9.16 (63.51–70.23)	62.57	13.57 (57.50–67.63)	64.97	11.36 (61–68.94)	
	POS 2	65.84	9.41 (62.39–69.29)	63	12.58 (58.30–67.70)	63.65	12.28 (59.36–67.93)	
	POS 3	67.74	10.50 (63.89–71.60)	64.03	12.91 (59.21–68.85)	66.15 ^Ψ^	18.90 (62.34–69.95)	
Right rotation	PRE	65.94	10.53 (62.07–69.80)	62.36	11.76 (57.44–66.23)	62.71	9.21 (59.49–65.92)	0.605
	POS 1	68.84	9.03 (65.53–72.15)	65.33 *	9 (61.97–68.69)	64.82	8 (62.03–67.62)	
	POS 2	68.19	9.69 (64.64–71.75)	65.60	7.88 (62.66–68.54)	66.41	7.82 (63.68–69.14)	
	POS 3	70.64 *	9 (67.35–73.94)	66	8.95 (62.66–69.34)	68.73 *	6.96 (66.30–71.16)	
Left rotation	PRE	65.90	9.79 (62.31–69.50)	62.60	11.34 (58.36–66.83)	64.85	10.85 (61.07–68.34)	0.350
	POS 1	67.74	9.61 (64.22–71.27)	65.43 *	12.13 (60.90–69.96)	67.53 *	9.11 (64.35–70.71)	
	POS 2	65.74	9.48 (62.27–69.22)	63.87	8.80 (60.58–67.15)	67.35	7.25 (64.82–69.88)	
	POS 3	68.65 ^Ψ^	9.33 (65.22–72.07)	65.37	8.79 (62.08–68.65)	71.47 *^Ψ^	9.14 (68.28–74.66)	
Right side bending	PRE	40	8.33 (36.94–43.06)	38.03	8.07 (35.02–41.05)	37.91	8.03 (35.11–40.71)	0.411
	POS 1	41.13	7.70 (38.30–43.95)	39.20	6.47 (36.78–41.62)	39.65	10 (36.16–43.14)	
	POS 2	41.65	6.54 (39.25–44.04)	41.50	9.56 (37.93–45.07)	37.94	7.04 (35.48–40.40)	
	POS 3	42.87	5.53 (40.84–44.90)	42.03 *	8.30 (38.93–45.13)	40.59 ^Ψ^	9.69 (37.21–43.97)	
Left side bending	PRE	44.29	10.55 (40.42–48.16)	44.57	9.39 (41.06–48.07)	44.23	6.79 (41.87–46.60)	0.378
	POS 1	45.55	9.97 (41.89–49.20)	44	8.54 (40.81–47.19)	46.18	7.93 (43.41–48.94)	
	POS 2	44.87	9.34 (41.44–48.30)	46.07	9.79 (42.41–49.72)	44.85	7.13 (42.37–47.34)	
	POS 3	46.77	9.36 (43.34–50.21)	47.70	8.88 (44.38–51.02)	48.24 *^Ψ^	7.46 (45.63–50.84)	

SD, standard deviation; CI, confidence interval; VAS, visual analogue scale; PRE, initial pre-intervention; POS 1, after the first intervention; POS 2, pre-intervention of the second week; POS 3, after the second intervention. * *p* < 0.05 compared to the baseline measurement. ^Ψ^ *p* < 0.05 compared to the previous measurement.

**Table 3:** PRE and POST VAS Measurements.

Variable	Measurement Time	Group 1 (Suboccipital Technique) (*n* = 31)		Group 2 (INYBI) (*n* = 31)		Group 3 (INYBI + Upper Cervical Manipulation) (*n* = 34)		*p* Value (Between-Groups Comparison)
		Mean	SD (95% CI)	Mean	SD (95% CI)	Mean	SD (95% CI)	
Flexion VAS	PRE	1.73	1.74 (1.09–2.37)	1.63	1.65 (1.02–2.25)	2.78	2.04 (2.07–3.49)	0.561
	POS 1	1.46	1.81 (0.80–1.34)	1.48	1.78 (0.81–2.14)	2.10 *	1.81 (1.46–2.73)	
	POS 2	0.90	1.21 (0.45–1.34)	0.98	1.56 (0.39–1.56)	1.70 *	1.85 (1.05–2.34)	
	POS 3	0.57 *	0.93 (0.26–0.91)	0.65 *^Ψ^	1.09 (0.24–1.06)	1.12 *^Ψ^	1.67 (0.54–1.70)	
Extension VAS	PRE	2.41	2.10 (1.63–3.18)	3.21	2.30 (2.35–4.07)	3.25	2.01 (2.55–3.95)	0.728
	POS 1	1.72 *	1.79 (1.06–2.38)	2.52 *	2.49 (1.59–3.45)	2.16 *	1.90 (1.50–2.83)	
	POS 2	1.31 *	1.60 (0.72–1.90)	1.85 *	1.50 (1.29–2.41)	2.09 *	2.05 (1.37–2.81)	
	POS 3	0.90 *	1.03 (0.52–1.28)	1.61 *	1.43 (1.07–2.14)	1.39 *^Ψ^	1.30 (0.94–1.85)	
R. rotation VAS	PRE	1.33	1.70 (0.71–1.95)	1.85	2.05 (1.08–2.61)	2.16	1.80 (1.54–2.79)	0.062
	POS 1	0.75 *	1.23 (0.30–1.21)	1.64	2.02 (0.88–2.40)	1.53 *	1.50 (1.00–2.05)	
	POS 2	1.07	1.42 (0.55–1.59)	1.36	1.30 (0.87–1.84)	1.76	1.81 (1.12–2.39)	
	POS 3	0.95	1.42 (0.43–1.47)	0.95 *	1.34 (0.45–1.45)	1.03 *^Ψ^	1.46 (0.52–1.54)	
L. rotation VAS	PRE	1.79	1.82 (1.13–2.46)	2.13	2.10 (1.35–2.91)	2.28	1.76 (1.66–2.89)	0.024
	POS 1	1.27	1.29 (0.80–1.75)	1.91	2.19 (1.10–2.73)	1.45 *	1.42 (0.96–1.95)	
	POS 2	1.36	1.72 (0.73–1.99)	1.26 *^Ψ^	1.44 (0.72–1.80)	1.48 *	1.41 (0.99–1.97)	
	POS 3	1.21	1.65 (0.61–1.82)	0.76 *^Ψ^	1.10 (0.35–1.17)	0.87 *^Ψ^	1.12 (0.48–1.26)	
Right SB VAS	PRE	2.99	1.93 (2.28–3.70)	3.06	2.60 (2.08–4.03)	3.78	2.14 (3.03–4.539	0.282
	POS 1	2.21 *	1.98 (1.48–2.93)	2.52	2.22 (1.69–3.35)	2.64 *	1.83 (2–3.28)	
	POS 2	2.22	2.11 (1.45–3)	2.04 *	1.75 (1.39–2.70)	2.65 *	2.13 (1.91–3.40)	
	POS 3	1.73 *^Ψ^	1.96 (1.01–2.45)	1.25 *^Ψ^	1.42 (0.72–1.78)	1.73 *^Ψ^	1.83 (1.09–2.37)	
Left SB VAS	PRE	3.18	2.24 (2.36–4)	3.2	2.56 (2.25–4.16)	3.58	2.07 (2.86–4.30)	0.474
	POS 1	2.48	2.30 (1.64–3.32)	2.75	2.49 (1.82–3.68)	2.39 *	1.81 (1.76–3.02)	
	POS 2	2.35 *	2.36 (1.48–3.21)	2.18 *	1.91 (1.46–2.89)	2.46 *	1.94 (1.79–3.14)	
	POS 3	1.68 *^Ψ^	2.16 (0.89–2.48)	1.50 *^Ψ^	1.80 (0.83–2.18)	1.59 *^Ψ^	1.63 (1.02–2.15)	

SD, standard deviation; CI, confidence interval; VAS, visual analogue scale; PRE, initial pre-intervention; POS 1, after the first intervention; POS 2, pre-intervention of the second week; POS 3, after the second intervention; R, right; L, left; SD, side bending. * *p* < 0.05 compared to the baseline measurement. ^Ψ^ *p* < 0.05 compared to the previous measurement.

**Table 4:** PRE and POST Algometry and NDI Measurements.

Variable	Measurement Time	Group 1 (Suboccipital Technique) (*n* = 31)		Group 2 (INYBI) (*n* = 31)		Group 3 (INYBI + Upper Cervical Manipulation) (*n* = 34)		*p* Value (Between-Groups Comparison)
		Mean	SD (95% CI)	Mean	SD (95% CI)	Mean	SD (95% CI)	
Right suboccipital	PRE	1.40	0.37 (1.26–1.54)	1.59	0.56 (1.38–1.79)	1.48	0.43 (1.33–1.63)	0.813
	POS 1	1.58 *	0.48 (1.40–1.76)	1.79 *	0.57 (1.57–2)	1.65 *	0.56 (1.46–1.85)	
	POS 2	1.57	0.44 (1.41–1.73)	1.71	0.56 (1.50–1.92)	1.70 *	0.64 (1.47–1.92)	
	POS 3	1.76 *^Ψ^	0.57 (1.56–1.97)	1.94 *^Ψ^	0.72 (1.67–2.21)	1.90 *^Ψ^	0.79 (1.62–2.17)	
Left suboccipital	PRE	1.31	0.49 (1.13–1.49)	1.57	0.51 (1.38–1.76)	1.46	0.38 (1.32–1.59)	0.678
	POS 1	1.53 *	0.50 (1.35–1.72)	1.80 *	0.52 (1.61–2)	1.62 *	0.49 (1.45–1.79)	
	POS 2	1.52	0.47 (1.34–1.69)	1.70	0.50 (1.52–1.89)	1.68 *	0.58 (1.47–1.88)	
	POS 3	1.67 *^Ψ^	0.54 (1.47–1.87)	1.91 *^Ψ^	0.68 (1.66–2.17)	1.86 *^Ψ^	0.68 (1.63–2.10)	
Right UT	PRE	1.35	0.44 (1.19–1.51)	1.55	0.42 (1.40–1.71)	1.51	0.47 (1.32–1.71)	0.574
	POS 1	1.43	0.44 (1.27–1.59)	1.70	0.51 (1.51–1.89)	1.51	0.66 (1.28–1.74)	
	POS 2	1.41	0.42 (1.26–1.56)	1.69	0.54 (1.48–1.89)	1.60	0.64 (1.38–1.83)	
	POS 3	1.55 ^Ψ^	0.46 (1.38–1.72)	1.85 *^Ψ^	0.63 (1.61–2.08)	1.79 *^Ψ^	0.83 (1.50–2.08)	
Left UT	PRE	1.43	0.36 (1.30–1.57)	1.56	0.38 (1.42–1.70)	1.48	0.47 (1.31–1.64)	0.317
	POS 1	1.51	0.37 (1.37–1.64)	1.65	0.41 (1.50–1.81)	1.55	0.50 (1.38–1.73)	
	POS 2	1.48	0.34 (1.35–1.61)	1.63	0.43 (1.47–1.79)	1.65	0.66 (1.42–1.88)	
	POS 3	1.60 ^Ψ^	0.47 (1.42–1.77)	1.75 ^Ψ^	0.47 (1.58–1.93)	1.81 *^Ψ^	0.71 (1.56–2.06)	
NDI	PRE	12.26	5.70 (10.17–14.35)	10.40	5.14 (8.48–12.32)	10.53	4.40 (8.99–12.07)	0.458
	POS	9.74 *	5.28 (7.81–11.68)	8.07 *	5.97 (5.84–10.29)	7.06 *	4.52 (5.48–8.64)	

SD, standard deviation; CI, confidence interval; VAS, visual analogue scale; PRE, initial pre-intervention; POS 1, after the first intervention; POS 2, pre-intervention of the second week; POS 3, after the second intervention; UT, upper trapezius; NDI, neck disability index. * *p* < 0.05 compared to the baseline measurement. ^Ψ^ *p* < 0.05 compared to the previous measurement.

## Data Availability

Data available on request due to ethical restrictions. The data presented in this study are available on request from the corresponding author. The data are not publicly available due to the Spanish Oganic Law of Protection of Personal Data and guarantee of digital rights.
